# Digital education about delirium for health care professional students: a mixed methods systematic review

**DOI:** 10.1186/s12909-024-05725-3

**Published:** 2024-07-15

**Authors:** Dympna Tuohy, Pauline Boland, Patrick Stark, Lana Cook, Tara Anderson, Heather E. Barry, Matt Birch, Christine Brown-Wilson, Emma Cunningham, James McMahon, Margaret Graham, Geoffrey M. Curran, Gary Mitchell, Jill Murphy, Audrey Tierney, Alice Coffey

**Affiliations:** 1 Department of Nursing and Midwifery, University of Limerick, Limerick, V94T9PX Ireland; 2 School of Allied Health, University of Limerick, Limerick, Ireland; 3 School of Nursing and Midwifery, Queen’s University Belfast, Belfast, BT9 7BL Northern Ireland; 4 Primary Care Research Group, School of Pharmacy, Queen’s University Belfast, 97 Lisburn Road, Belfast, BT9 7BL Northern Ireland; 5 Centre for Public Health, Institute of Clinical Sciences, Queen’s University Belfast, Block B, Belfast, BT12 6BA Northern Ireland; 6 Center for Implementation Research, University of Arkansas for Medical Sciences, Little Rock, AR USA

**Keywords:** Delirium, Digital education, Health care professionals, Pre-registration, Students

## Abstract

**Background:**

Competence in delirium care begins with pre-registration education for health care professionals. Although a common complication for hospitalised patients, delirium is avoidable and reversible. Delirium requires early recognition in person-centred care. Students need to learn how to identify and effectively care for ‘at risk’ patients.

**Aim:**

To identify and examine literature on how pre-registration health care professional students are prepared to recognise, assess, and deliver interventions to prevent delirium in practice, using digital/web based educational interventions.

**Method:**

Mixed methods systematic review with narrative synthesis. A protocol was registered with PROSPERO. The review questions and search strategy were guided by the Population, Phenomena of Interest, Context (PICo) framework. The PRISMA framework guided the screening, data extraction and analysis. Database searches (MEDLINE, Web of Science, Embase, CINAHL, Cochrane Central Register of Controlled Trials, PsycINFO & Scopus) were undertaken in April 2023 for publications from 2012 to 2023. Covidence software [30] was used to extract and manage the data. Quality appraisal was guided by the Crowe Critical Appraisal Tool (CCAT) [31].

**Findings:**

Ten papers were included: mixed methods (2), qualitative (1) and quantitative (7*)*. Medical students were the most studied group (*n* = 5), followed by student nurses (*n* = 4) and mixed nursing and medical students (*n* = 1). Length of learning experience varied from 12 min virtual reality (VR) to a two-week ‘geriatrics’ elective. Learning was enhanced by player autonomy, engagement, safety, applicability, choices, multiple perspectives and moral reasoning opportunities.

**Discussion:**

Digital programmes should be visually appealing, interactive with opportunities for practice and timely appropriate feedback.

**Supplementary Information:**

The online version contains supplementary material available at 10.1186/s12909-024-05725-3.

## Introduction and background

Delirium has been described as “a clinical state characterized by a combination of features defined by diagnostic systems such as the DNM-5” [1] (pg 1021). These features include disturbances in attention and awareness, short onset of hours/a few days, fluctuating in severity with cognitive disturbance and arise from “another medical condition, substance intoxication or withdrawal…or exposure to a toxin, or…multiple etiologies” [1](pg 1021). Delirium can be distressing for the person experiencing it [2–4].

Although it can be avoidable and reversible, it is the most common complication for hospitalised older people and its prevalence rises with age [5, 6]. Delirium occurs in an estimated 30% of inpatients admitted to a medical ward, 50% of older adults in a surgical ward and 60% of residents living in a care home setting [5]. There are several predisposing factors for delirium including pre-existing impairment (cognitive impairment, dementia, functional impairment such as frailty, vision, hearing, physical), depression and co-morbidities [7, 8]. Precipitating factors comprise medication, care procedures, sleep deprivation, iatrogenic events, pain, environmental factors [8, 9].

Unfortunately, delirium is frequently underdiagnosed [10, 11] and awareness and reporting procedures need to be improved [11, 12]. Early recognition of delirium is essential (Health Service Executive) [13] for patient care and it is crucial that health care professionals can identify delirium, to assess, plan, implement and evaluate effective person centred care [14, 15]. It is imperative that competence in delirium care begins in pre-registration professional health care education [16]. It is also important that students learn to identify patients who are ‘at risk’ of delirium and know how to effectively care for them.

Several systematic reviews have examined educational interventions aimed at improving recognition and assessment of delirium [17–19]. However, there is a lack of studies that synthesise the evidence on how delirium education is taught and understood by professional health care students [20, 21]. In terms of pedagogy, various theoretical approaches can inform and direct how online learning can be effectively leveraged to not just replace, but enhance student learning and related outcomes for health care professionals. Sahu et al*.* [22] argue that there are three main theoretical approaches to learning and that these need to be considered when developing online educational programmes. Sahu et al*.* [22] define behaviourism (clear deadlines, reward for effective learning), cognitivism (use of problem-based learning to engage existing knowledge and problem-solving to deal with multi-faceted issues) and constructivism (where learner constructs meaning about a phenomenon through activities). The approaches all have resonance for delirium education, where students can role-play and practice as a health care professional, able to interpret key delirium signs and engage with clear clinical reasoning about options for care. Peer learning can be maximised using carefully designed online learning approaches [23] while interprofessional learning and related collaboration is key to delirium education [24] and can be effectively delivered through online methods [25].

The aim of this mixed method systematic review was to examine the literature on how pre-registration healthcare professional students are prepared to recognise and assess delirium and deliver interventions to prevent delirium in practice using digital or web based educational interventions. Relevant modes of delivery and related theories of education (where present) were collated.

## Method

The intended outcome of this review is to inform the future design of a digital educational tool for delivering delirium education. Therefore, it was deemed important to review both qualitative and quantitative studies using a mixed methods systematic literature review design. The research team have topic (health care disciplines and gerontology) and systematic review methodological expertise. A review protocol was developed to guide the mixed methods systematic review by complying with the Preferred Reporting Items for Systematic reviews and Meta-Analyses (PRISMA) framework [26]. It described the review question, review types, eligibility criteria, search strategy, data extraction and analysis process [26, 27]. The protocol was registered on PROSPERO register (registration number: CRD42023422411) in May 2023 [28]. This mixed methods systematic review is reported in line with the Preferred Reporting Items for Systematic Reviews and Meta-Analyses Extension for Scoping Reviews [26]. The PRISMA guidelines checklist provides a “standard peer accepted methodology” [27] (page 3). Following the PRISMA Framework provides structure and a clear audit trail of the systematic literature review process [26]. As this was a mixed methods systematic review, there is no requirement for an Ethics Approval declaration nor a Consent to Participate declaration.

### The review questions

1.What is the impact of current digital or web based delirium education programmes on health care professional students’ learning and practice in higher education?

2.What can be understood about the nature and effectiveness of digital education programmes for professional health care students learning about delirium car

### Search strategy

The literature search was undertaken in April 2023.The Population, Phenomena of Interest, Context (PICo) framework [29] was used for this review: the Population was ‘pre-registration health care professional students’, the Phenomenon of interest was ‘digital or web based delirium education’ and the Context was ‘tertiary education’. The search strategy identified keywords and search terms.

Boolean operators were used to refine the search strategy. The Boolean operator of AND has the function to narrow the search, OR has the function to broaden, NOT has the function to make more specific, and asterisks were used to capture word stems and /or variations in spelling**.** Keywords and search terms are outlined in Supplemental file 1. MEDLINE, Web of Science, Embase, CINAHL, Cochrane Central Register of Controlled Trials (CENTRAL), Cochrane Database of Systematic Reviews, PsycINFO and Scopus databases were searched for publications from 2012–2023. Focusing on a ten-year period, from 2012 to 2023, ensured the inclusion of recent research studies and reflects the evolving landscape of digital education and delirium care. The inclusion and exclusion criteria are identified in Table 1. Covidence software [30] was used to extract and manage the data.

**Table 1:** Inclusion and exclusion criteria

Criteria	Inclusion	Exclusion
Time frame	January 2012 to April 2023	Before January 2012
Language	English	Non English
Type of literature	Research studies (qualitative, quantitative, mixed design)	Grey literature Commentaries Letters Opinion pieces
Population	Pre-registration health care professional students in tertiary education, including students from undergraduate/postgraduate pre-professional health and social care registration programmes: nursing, midwifery, medicine, physician assistants, physiotherapy, occupational therapy, speech and language therapy, pharmacy, social work, social care, psychology, paramedics, nutrition, dietetics, dietitian	Registered practitioners in health and social care professions Junior doctor/residency programmes Hospital based programme Health and social care assistants
Intervention	Digital or web-based education on delirium for pre-registration health care professional students	Delirium education which is not wholly digital or web based (e.g. blended delivery) Delirium education which is facilitated face to face in a classroom
Context	Tertiary education	Primary education Secondary education

### Screening

**Fig. 1:**
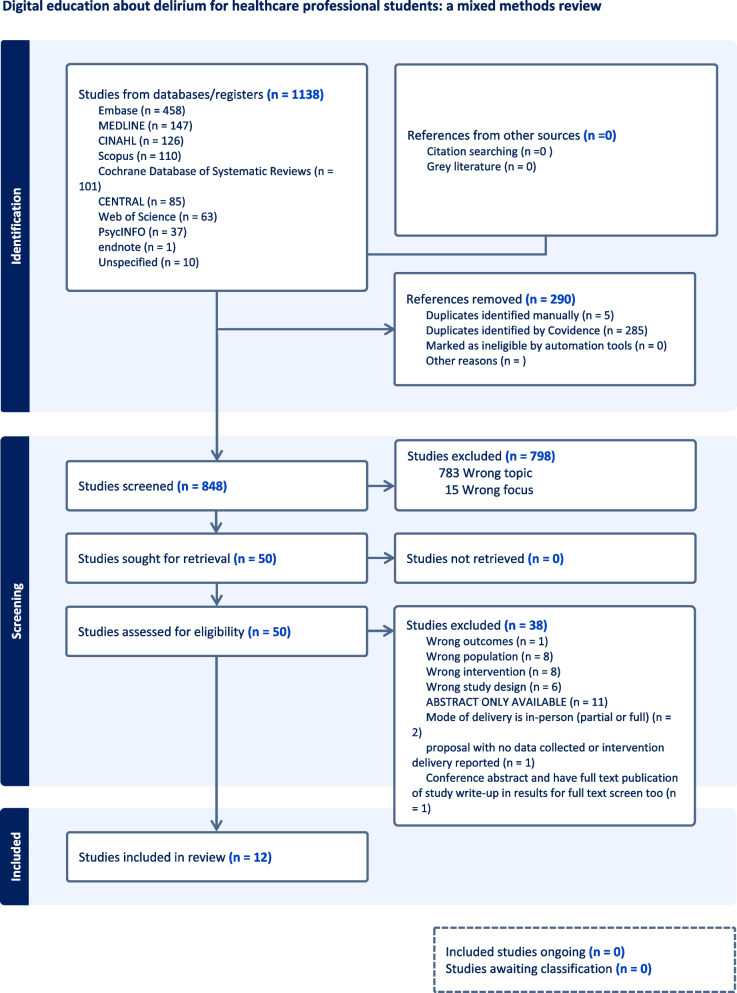
Prisma flow diagram

The search yielded 1138 citations. All references were imported for screening to Covidence software [30]. References were from Embase (458), MEDLINE (147), CINAHL (126), Scopus (110), Cochrane Database of Systematic Reviews (101), CENTRAL (85), Web of Science (63), Psychinfo (37), endnote (1) and reference lists (10). Duplicates were removed (290). Screening was undertaken against the inclusion/exclusion criteria to determine eligibility (Table 1). Titles and abstracts of papers (848) were independently screened by two reviewers from the research team (in total, four team members, working in different paired combinations) (PB, LC, PS, DT). Conflicts were resolved by discussion with a third reviewer (one of the four team members, PB, LC, PS, DT) and 798 papers were excluded. Full text screening of the remaining 50 papers were independently screened by two reviewers with conflicts resolved by a third reviewer (PB, LC, PS, DT). The reasons for excluding 38 papers were: abstract only available (11); wrong intervention (8); wrong population (8); wrong study design (6); mode of delivery is in-person (partial or full) (2); conference abstract (1); wrong outcomes (1), and proposal with no data collected or intervention delivery reported (1). This resulted in 12 papers remaining for quality appraisal. Results are presented in a Preferred Reporting Items for Systematic Reviews (PRISMA) flow diagram (Fig. 1) [26]. The PRISMA framework guided the search strategy, screening, data extraction and analysis. The use of the PRISMA framework supports high quality systematic literature review [27]. Quality appraisal was undertaken using the Crowe Critical Appraisal Tool (CCAT) [31].

### Data evaluation

The remaining 12 papers were critically appraised using the Crowe Critical Appraisal Tool (CCAT) [31]. This objective appraisal of the quality of the papers [32] was undertaken by three reviewers (PB, PS, DT). There are eight categories within the CCAT tool and each category is scored using a six point scale (from 0–5); there is an overall maximum of 40, which is then converted into a percentage [31]. A pilot test of appraisal was undertaken of three papers to support consistency between raters. Two of the reviewers (PS, DT) independently appraised these papers and then compared and discussed the results for consistency. Thereafter appraisals were individually completed by three reviewers from the team members (duplicate appraisals were not undertaken). The scores of the appraised papers ranged from 25% (10/40) to 88% (35/40). Two papers (scored 25% and 28%) were excluded at this point due to lack of rigor (e.g. lacked detail on data, data collection methods, sample, ethics, potential bias and limitations) and ten (scores ranged from 65 to 88%) were included in the review. Determining a minimum score was informed by agreeing to examine the individual scores per category plus the overall score for even distribution and this being the case, accept a threshold of 60%. These research papers and their CCAT scores are presented in the data extraction table (Supplemental file 2).

### Data extraction

Data extraction was undertaken for the component parts of the qualitative and quantitative studies [33]. Three team members (PB, PS, DT) extracted the 1) intervention title and description; 2) setting; 3) outcomes measures; 4) results; 5) facilitators and barriers. The intervention details and setting were examined to explore the educational context of delivery, the professional backgrounds of health care students who had undertaken such educational programmes, and the design of digital/ web based delirium education programmes. Outcomes were identified to increase our understanding of how success is measured in health-based delirium education. The facilitators and barriers were examined to determine implementation factors which should be considered in future programme design (the intended next phase of this research).

#### Findings

This review assessed and narratively synthesised the literature on how pre-registration health care professional students are prepared to recognise, assess, plan, deliver and evaluate care interventions to prevent delirium in practice using digital or web based educational interventions. The findings are presented as a narrative synthesis of the data from ten papers that met the review criteria. The findings are presented under the following headings: study characteristics, types of digital education programmes, facilitators and barriers associated with such programmes.

#### Study characteristics

There are ten papers arising from five countries (Netherlands *n* = 4, USA *n* = 2 Canada, *n* = 1 Japan *n* = 2 and UK *n* = 1). Papers were published between 2012 – 2022, with most publications between 2020 – 2022. All four papers from Buijs-Spanjers et al. [34–37] appear to be based on the same sample and study timeframe. Both papers from Japan [38, 39] reported on the same cohort of students and data collected about their engagement with virtual reality (VR)depicting delirium-like hallucinations*.* Overall, medical students were the most studied group for this type of education (*n* = 5), followed by student nurses (*n* = 4) and mixed group nursing and medical students (*n* = 1). There were no studies with allied health students. The length of the online learning experience varied from 12 min VR [38, 39] to a two-week multi-modal geriatric care elective [40].

The research methods described in the papers were varied. They included: one qualitative descriptive [36], two mixed methods design [35, 37] and seven quantitative studies. These quantitative studies were two observational cohort studies [38, 39]; one descriptive account/case study of an optional elective designed for online delivery [40]; two pre and post design studies (one which investigated the impact of a podcast on nursing care for someone with delirium [21] and the second one was a quasi-experimental pre-post-test design [41]; one quantitative three arm randomised control trial [34] and one controlled cohort study [42] that compared different year groups where one group had a live lecture (2004—2005) vs another group who had with an online curriculum (2005—2006). The in-person lecture and online learning were mandatory at time of administration over a four week clinical clerkship (clinical experience) in Geriatrics and Home Medical Care [42].

#### Types of digital education programmes

There were different types of programmes ranging from: online serious game, online lecture, e-learning activity, Oculus Quest VR programme, two week multi-modal experience and a podcast. Oculus Quest was an all-in-one virtual reality headset.

Four papers wrote about the same online serious game approach [34–37] and each paper described different aspects of research undertaken on one serious game programme. Buijs-Spanjers et al. [34] described the online serious game as an interactive learning strategy whereby participants (third year Bachelor Medical Sciences students, *n* = 156) were actively involved in playing a video game (video simulation). It was based on the premise of experiential learning whereby people learn by ‘experiencing delirium’ and by doing (providing care). In this situation, one cohort, the game group played the delirium experience video game. The video focused on two perspectives, the patient experiencing delirium and the health care provider's perspective. The game lasted 20 min and there were scenarios for day and night (‘four days/nights’). The second group (control D) watched a video with explanations on delirium and a patient's experiences of delirium. The last group (control A) watched a video on healthy ageing. According to Buijs-Spanjers et al. [35] online serious gaming comprises two versions, one ‘dark’ with more moral reasoning elements and the other ‘normal’ version. During the game, players (third year Bachelor Medical Sciences students, *n* = 157) experienced ‘four days’ as a caregiver and then the corresponding nights as the patient who was experiencing delirium during a 20 min period. There were different actions available regarding care. Depending on actions chosen, delirium could vary by severity and there could be different options the following day. Feedback was given to the players at the end of the exercise on how their care could improve. Players then switched role to experience the patient’s perspective.

Buijs-Spanjers et al. [36] describe serious games as learning environments with an interactive application which allows the user to 'play the game' (takes 20 min) but its purpose is more than just playing, as it aims to educate. This delirium experience game focused on the narrative aspect of an interactive educational game. The narrative was about an older person receiving hip surgery and the health care professional who provided care. The experiential aspect of this enabled students (nursing students, *n* = 7 and medical students, *n* = 9) to learn from their experience of trying to care for the patient in the 'game'. The narrative approach promoted a humanistic approach to care and aimed to create empathy. Through immersion in the game and through individual tailored feedback, students’ attitudes could be influenced and ultimately could result in better understanding, communication, knowledge of patients’ care needs and care delivery to patients experiencing delirium [36]. Additionally, Buijs-Spanjers et al. [37] described serious games as educational interventions which promote experiential learning within a safe environment where learners (third year Bachelor Medical Sciences students, *n* = 160) can practice and there is no risk to real patients. There was active student involvement and learner autonomy was promoted in that they could choose their own options of either good or poor care within the game. The aim was that learners will be able to apply this new knowledge and skill to future clinical practice.

The second type of programme, identified in the review was an online lecture for 4th year medical students. The study’s aim was to design, deliver and test if an online curriculum on delirium diagnosis, evaluation and management would be as effective as a live lecture [42]. Two student cohorts were compared, those who had live lectures with those who had online only (the programmes were mandatory). This was delivered during a four week clerkship in Geriatrics and Home Medical Care. This programme centred on the online delivery of lecture material with interwoven multiple choice questions (MCQs), based on an already introduced case study on a patient with delirium and her family [42]. Two modules were delivered online and were undertaken in the students’ own time during the four week period. These were on 1) epidemiology, risk factors and diagnosis and 2) evaluation, management and prevention. The video clips included mock charts such as progress notes from previous clinic visits and past health history (e.g. medical, functional status and social). An interactive aspect of the study included asking questions on the slides for students to answer (students’ answers to these questions were not graded). Upon completion students took a 10 question quiz and needed 7/10 correct answers to achieve 5% for this specific rotation in their medical education [42]. Results indicated that online learning was as successful as the traditional lectures on teaching delirium evaluation and management. Interestingly, online learning students demonstrated better ability to suggest options for patient management, especially when it came to identifying the problem as delirium. It should be noted that no measures of student engagement with the two teaching methods were reported in this study.

The third programme type was an e-learning activity developed by Kalogirou et al. [41]. This was a cognitive impairment e-learning activity which comprised simulated case studies using videos, games and scenarios. The philosophical underpinning for this activity was the social learning theory and was demonstrated through positive nursing practice modelling. The programme was delivered as an asynchronous online learning activity in a foundational course in autumn 2019 and could be completed anytime between September–November on Google sites with pass/fail marks. There were eight sections covering cognitive impairment, delirium, depression, dementia and responsive behaviour, as well as a scenario and two sections which provided additional resources. Students completed a 12 item true/false quiz and a feedback survey. Results indicated that students' knowledge about cognitive impairment in older people increased following the e-learning activity. The qualitative student feedback identified the activity as both helpful and as an enjoyable way to learn and provided suggestions for improvement.

Two papers [38, 39] described the fourth programme type which was a VR programme. Both papers seem to be reporting on the same programme and same research study. The programme was a ‘Postoperative Delirium Experience System for Nursing Student Education Using VR and HMD’. HMD refers to head-mounted display. Eighteen students participated in the study (six from each of the following grades of a nursing major, second, third and fourth). The programme utilised an Oculus Quest 2 head-mounted display with VR images (changing every two minutes) of a 12-min simulated experience of postoperative delirium in an ICU setting (ICU training room in the university). Students were able to experience delirium from the patient’s perspective such as hallucinations of cockroaches on the ceiling, the ceiling approaching them, a person wearing a protective suit and attacking soldiers. Psychological and physiological changes were assessed, and students completed a pre and post questionnaire and had their autonomic nervous system measured whilst watching the VR so as to understand their stress levels. Biosensor heart rate variability, BITalino HeartBIT and Open Signals software were used for data acquisition of physiological response. It was extrapolated that having experienced the delirium simulation, participants were more likely to be empathetic towards patients experiencing delirium [38, 39].

The fifth programme was described by Michener [40] as a two week multimodal virtual geriatric care elective entitled “The 5 M’s and More: A New Geriatric Medical Student Virtual Curriculum During the COVID-19 Pandemic”. This was an optional elective designed for online delivery. The students were 'Clerkship' (clinical placement) students and were in either their second, third or fourth year of medical school. Each day of course instruction included a combination of short online didactics (presented live or prerecorded), readings, podcasts, interactive cases, discussion board posts, and virtual group discussion. Canvas (learning management system) was used to organise the course and assignments were based on 5 Ms (medications, mobility, mind, multicomplexity and what Matters most). Open-ended course feedback highlighted new appreciation for geriatric principles, especially the 5 M’s framework and polypharmacy/deprescribing. Feedback also included a range of opinions regarding different teaching modalities, with one recurring theme being a preference for small group discussion. Furthermore, the blend of synchronous and asynchronous learning supported the different learning preferences of the students.

The last and sixth programme was a 60 min podcast with case studies which had been co-designed with people who had experience of delirium [21]. A MP3 podcast was made available via Canvas and was embedded with two real-life stories from the experiences of the podcaster. These stories focused on a hospitalised patient living with hyperactive delirium (introduced approximately 1 min into the podcast) and a care home resident living with persistent hypoactive delirium (introduced approximately 40 min into the podcast). These narratives described key facts about the different types of delirium and were threaded throughout the podcast to support student understanding of the topic. Within the podcast, were three core elements namely defining delirium, recognising and managing delirium. This podcast was made available for 320 nursing students for 4 weeks throughout May and June 2020 and was a mandatory part of their education [21].

#### Barriers

Barriers to learning within the digital education programmes were identified. Greater clarity was required at times, for example some students found it difficult distinguishing between the narrative and aspects of the serious game [37]. Narrative(s) provided key facts about types of delirium to facilitate student understanding of the topic. Aspects of the game related to the choices that students could make relating to patient care within the game(s), feedback and suggestions given to students, the time given to complete the game and repetition within the videos. Students wanted more clarity on the type of feedback provided [36, 37] whereas others would have like more written feedback on why certain choices were incorrect [37] and suggestions as to how knowledge and practice could be improved. The time required to complete the programme varied between students, for example some students found it too long and so the optimal amount of time varied depending on the student. Furthermore, within the cognitive impairment e-learning activity [41] there was some repetition and the students found that several videos conveyed the same message. From a teaching staff perspective, provision of feedback was time consuming [40].

Students in the study by Buijs-Spanjers’s et al. [35] study experienced some technical difficulties for example the ‘plug in’ (a ‘plug in’ is a software component which adds a particular feature to an existing computer program) did not work for all and it necessitated students working in pairs which may have influenced their experience and resulted in an educational experience that was not solely delivered online. Chao et [42] indicated that the gains of online over in-person were relatively small and ultimately it is unclear if a combination of both online/in-person would have been better than either method delivered alone. From the perspective of whether universality could be achieved, this is unclear as it is not known how well videos will age and/or whether all games will suit students from different countries and different cultural contexts. Furthermore, cost may be an issue in relation to the use of VR headsets, as well as the need for ongoing topic updates and training [38, 39]. It may also be challenging to elicit and replicate the patients’ actual experiences [38, 39].

#### Facilitators

Several facilitators of learning through digital means were identified, including feedback, realism, autonomy and having multiple perspectives. Receiving both direct and indirect feedback on their performance supported student learning, for example they were able to directly see the consequences of their actions or inaction within the activity [36, 37]. Furthermore, receiving real-time student feedback enabled educators to make changes to assessment type e.g. from written assignment to small group session [40]. Learning was enabled in several ways such as having both synchronous and asynchronous sessions and this helped with students’ different learning styles [40]. Small group session learning and having clear examples and clear presentation of material facilitated learning and helped develop knowledge and confidence about delirium for student nurses [40]. Student autonomy was supported through being able to be self-directed, self-paced, being able to revisit material [41]. Freedom of choice seemed to be an important facilitator of learning in serious gaming [37]. Learning in a safe online environment was important to students [34]. The ability to learn in a safe environment where one can make mistakes and learn from them was important.

The realism of the activity also supported learning by facilitating engagement through interactive and experiential learning, which helped students to develop insights into the experience [36, 37]. Furthermore, having time limitations also added to the reality of having to make decisions within situations akin to clinical practice [37]. This realism was further enhanced by incorporating the perspectives of the patients and carers by using videos of hallucinations as these helped students develop empathy with delirious patients, while having an outline of the nurse experience helped develop empathy for staff in such care situations [35, 38, 39]. Ultimately, the studies indicate that digital programmes proved to be equivalently successful to traditional lectures on delirium evaluation and management, with students showing greater ability to suggest options for patient management, especially when it came to identifying the problem as delirium when using online learning [42].

## Discussion

The aim of this mixed methods systematic review was to identify and examine literature on how pre-registration health care professional students are prepared to recognise, assess, and deliver interventions to prevent delirium in practice, using digital/web based educational interventions. This review has identified several principles which enhanced online learning on the topic of delirium, which included player autonomy whereby students need to choose between care options and provided them with moral/ethical reasoning opportunities. They also include having the opportunity to actively engage in interactive, realistic experiential learning in which they are exposed to a range of severity of delirium states with related treatment options and multiple perspectives such as the perspective of the nurse/allied health professional as well as patient and/or caregiver. Furthermore, the principle of having a safe environment in which to make mistakes and learn from these and being provided with feedback on how to improve reasoning on care options was present in the online programmes.

From our findings, the evidence-base in this area is narrow but some preliminary observations can be made. First, this mode of teaching and learning is acceptable to health care students given it is clinically applied but safe, particularly when interactive and with regular, tangible feedback, students are able to understand, consistent with behaviourism and constructivism approaches embedded within teaching and learning online [22]. Building empathy appears to be one key impact of this mode of learning [38, 39] as well as enhanced moral reasoning [34–37], where students construct an understanding of what it might be like to have delirium and therefore take a compassionate and more informed approach to care. This construction of meaning from a learning experience [43] would be key to any future developments in delirium education and related evaluation.

Ensuring an interactive element is prioritised within the learning experience is vital for success, both with other students online and with facilitators, and  appears to be integral to designing effective online learning about delirium. This is similar to studies informed by cognitivism (making sense of a phenomenon by discussing with others and drawing on existing knowledge) [44]. One study in this review found that online delivery proved to be equivalently successful to traditional lecture on delirium evaluation and management, with students learning via online methods showing better ability to suggest options for patient management, especially when it came to identifying the problem as delirium and related management options [42].

The importance of safe practice of skills with high stakes (e.g. getting care decisions ‘wrong’ and seeing the consequences in virtual rather than real-world environments) and visual appeal of online materials were seen as pivotal to maximising the online learning for students on delirium care. Essentially, valuable experiential learning [45] without the risks and pressure of the actual clinical environment, can be offered through well-designed and executed online programmes of study. Furthermore, feedback on performance was important, given that  feedback is a core driver of all learning [46, 47] this is not surprising. Individualised (to a student) as well as general feedback seems to be part of existing models of online learning.

### Strengths and limitations

This review, as with all reviews, is limited by the underpinning data and methodology in this area. The number of articles which met our inclusion criteria were relatively small, though this was not unexpected. While delivering this learning via digital means enhanced outcomes (where this was measured) there is very little comparison between the two modes of learning. Interprofessional learning and collaboration is seen as key in post-registration education on delirium identification and management [24] however we sourced only one study where students had an interprofessional element to their learning.

This paper is the first to collate what is known about digital learning for health care professional students for people with delirium. This information will form part of the foundation for informing a novel, online based education programme for health care professionals. The introduction of serious gaming to health care education offers a novel approach to theory supporting health care professional students, that of play and entertainment to support motivation and engagement in complex reasoning [48].

### Recommendations

Opportunity for practice and being visually appealing would enhance further engagement. Principles to underpin online learning for delirium identification and management could include: 1) Autonomy, where students have the opportunity to be self-directed in a supportive online environment, self-paced and able to revisit material, 2) Freedom of choice seems to be an important facilitator of learning in serious gaming and 3) Clear and engaging presentation of material facilitates learning on knowledge and confidence about delirium.

For learning to be authentic, existing evidence presented in this review has shown that both carer and family member perspectives need to be presented in a compelling and clear way. Other key recommendations from the work presented here include that online learning for delirium education is visually appealing, that interprofessional and collaborative learning is at the heart of any future work given the lack of existing literature including these two well recognised pedagogical approaches in health care education [44].

## Conclusion

It is imperative that pre-registration health care professional students are prepared to recognise, assess and deliver interventions to prevent and/or ameliorate delirium in practice. This mixed methods review examined the literature on using digital or web based educational interventions to teach students about delirium. For such educational programmes to be effective it is important that they are interactive, engage the learners and provide opportunities for practice. It is also important that students are afforded opportunities to understand and appreciate the perspectives of the patient, their families and other health care professionals. Lastly, feedback on knowledge and performance needs to be provided to be timely and positive.

### Supplementary Information

Supplementary Material 1.

Supplementary Material 2.

## Data Availability

Data is provided within the manuscript or supplementary information files.
