# Supplementary material for: Digital education about delirium for health care professional students: a mixed methods systematic review
Source: BMC Med Educ. 2024 Jul 15;24:762. doi: 10.1186/s12909-024-05725-3 (PMC11247797; doi:10.1186/s12909-024-05725-3)
Supplement: Supplementary file 1 — Supplementary Material 1. [file 12909_2024_5725_MOESM1_ESM.docx]

| **Supplemental file 1: Search Terms** |  |  |  |  |
| --- | --- | --- | --- | --- |
| **Search terms 1** |  |  |  |  |
| **PICO Element** | **Key terms** | **Emtree term** | **Synonyms / free text keywords** | **Truncation/wild card use** |
| ***Participants (P): Pre-registration health profession students*** | **Health care profession students** |  | nursing student* OR student nurse*  OR midwife$ student* OR student midwi$  OR student doctor* OR medical student* OR student* of medicine OR med* student OR student physician assistant* OR physiotherap* student*  OR occupational therap* student* OR Speech and Language Therap* student* OR pharmacy student*  OR Social work student*  OR Social Care student* OR student psycholog$ OR student paramedic* OR paramedic student* OR nutrition student* OR student nutrition$  OR Dietetics student* OR dietetics student* OR student dietetic* OR Dietitian student* OR student dietitian* | va |
| ***0*** | **1) Digital or web-based educational interventions** | Digital technology OR Virtual Reality, Educational OR computers, digital OR personal digital assistant OR internet-based intervention OR Digital Learning OR E-Learning or Online Learning or Web-Based Learning OR Online Education or Remote Teaching or Remote Learning or Mobile Learning or Virtual Learning or Serious Game or Gamification or Podcast or Web-Based App or App or MOCC |  |  |
|  | **2) Delirium** | Delirium OR Subacute delirium  OR delirium of mixed origin OR Sepsis Associated delirium  OR emergence delirium OR post-operative delirium OR anesthesia induced delirium OR postoperative delirium  OR acute confusion OR Confusion State or Confusional State or Acute Confusional State OR Acute Brain Dysfunction OR Hypoactive delirium OR Hyperactive delirium OR Mixed delirium OR Alcohol withdrawal delirium OR ICU psychosis OR intensive care psychosis OR delirium tremens OR alcoholic delirium OR Delirium, Dementia, Amnestic, Cognitive Disorders OR delirium management OR delirium assessment OR icu syndrome OR intensive care syndrome OR post-intensive care syndrome OR altered mental status OR confusion |  |  |
| ***Context: Undergraduate and postgraduate programmes at third-level education*** |  | Education OR Educational Activities OR Education, Nursing OR Nursing Education OR Education, Nursing, Baccalaureate OR Education, Nursing, Graduate OR Graduate Nursing Education OR Midwifery Education OR Midwifery, Graduate OR Education, Pharmacy OR Pharmacy Education OR Education, Dental OR Dental Education OR Education, Medical OR Medical Education OR Undergraduate Medical Education OR Education, Medical, Undergraduate OR Education, Undergraduate Medical OR Interprofessional Education OR Allied Health Occupations OR Pre-registration education   OR mentors OR teaching OR teaching methods OR Education, Professional  OR teach$ OR education$ OR learn$ OR course$ |  |  |
|  |  |  |  |  |
| **Search terms 2** |  |  |  |  |
| **PICO Element** | **Keyword** | **Emtree term** | **Synonyms / free text keywords** |  |
| ***Participants (P): Pre-registration health profession students in tertiary level education*** | **Health care profession students** | Education OR Educational Activities OR Education, Nursing OR Nursing Education OR Education, Nursing, Baccalaureate OR Education, Nursing, Graduate OR Graduate Nursing Education OR Midwifery Education OR Midwifery, Graduate OR Education, Pharmacy OR Pharmacy Education OR Education, Dental OR Dental Education OR Education, Medical OR Medical Education OR Undergraduate Medical Education OR Education, Medical, Undergraduate OR Education, Undergraduate Medical OR Interprofessional Education OR Allied Health Occupations OR Pre-registration education   OR nursing student* OR pharmacy student* OR medical student* OR occupational therap* student* OR physiotherap* student*  OR Speech and Language Therap* student* OR Social work student* OR Social Care student* OR Dietetics student* OR Dietitian student*  OR mentors OR teaching OR teaching methods OR Education, Professional  OR teach$ OR education$ OR learn$ OR course$ | nursing student* OR student nurse*  OR midwife$ student* OR student midwi$  OR student doctor* OR medical student* OR student* of medicine OR med* student OR student physician assistant* OR physiotherap* student*  OR occupational therap* student* OR Speech and Language Therap* student* OR pharmacy student*  OR Social work student*  OR Social Care student* OR student psycholog$ OR student paramedic* OR paramedic student* OR nutrition student* OR student nutrition$  OR Dietetics student* OR dietetics student* OR student dietetic* OR Dietitian student* OR student dietitian* | nursing student* OR student nurse*  OR midwife$ student* OR student midwi$  OR student doctor* OR medical student* OR student* of medicine OR med* student OR student physician assistant* OR physiotherap* student*  OR occupational therap* student* OR Speech and Language Therap* student* OR pharmacy student*  OR Social work student*  OR Social Care student* OR student psycholog$ OR student paramedic* OR paramedic student* OR nutrition student* OR student nutrition$  OR Dietetics student* OR dietetics student* OR student dietetic* OR Dietitian student* OR student dietitian* |
| ***Intervention/phenomena of Interest (I): Digital or web-based educational interventions on delirium*** | **Digital or web-based educational interventions** | Digital technology OR Virtual Reality, Educational OR computers, digital OR personal digital assistant OR internet-based intervention OR Digital Learning OR E-Learning or Online Learning or Web-Based Learning OR Online Education or Remote Teaching or Remote Learning or Mobile Learning or Virtual Learning or Serious Game or Gamification or Podcast or Web-Based App or App or MOCC |  |  |
| ***Context: Delirium*** | **Delirium** | Delirium OR Subacute delirium  OR delirium of mixed origin OR Sepsis Associated delirium  OR emergence delirium OR post-operative delirium OR anesthesia induced delirium OR postoperative delirium  OR acute confusion OR Confusion State or Confusional State or Acute Confusional State OR Acute Brain Dysfunction OR Hypoactive delirium OR Hyperactive delirium OR Mixed delirium OR Alcohol withdrawal delirium OR ICU psychosis OR intensive care psychosis OR delirium tremens OR alcoholic delirium OR Delirium, Dementia, Amnestic, Cognitive Disorders OR delirium management OR delirium assessment OR icu syndrome OR intensive care syndrome OR post-intensive care syndrome OR altered mental status OR confusion |  |  |
